# Global Stability of Vector-Host Disease with Variable Population Size

**DOI:** 10.1155/2013/710917

**Published:** 2013-06-09

**Authors:** Muhammad Altaf Khan, Saeed Islam, Sher Afzal Khan, Gul Zaman

**Affiliations:** ^1^Department of Mathematics, Abdul Wali Khan University, Mardan, Khyber Pakhtunkhwa, Pakistan; ^2^Department of Computer Sciences, Abdul Wali Khan University, Mardan, Khyber Pakhtunkhwa, Pakistan; ^3^Department of Mathematics, University of Malakand, Dir, Pakistan

## Abstract

The paper presents the vector-host disease with a variability in population. We assume, the disease is fatal and for some cases the infected individuals become susceptible. We first show the local and global stability of the disease-free equilibrium, for the case when *R*_0_ < 1. We also show that for *R*_0_ < 1, the disease free-equilibrium of the model is both locally as well as globally stable. For *R*_0_ > 1, there exists a unique positive endemic equilibrium. For *R*_0_ > 1, the disease persistence occurs. The endemic equilibrium is locally as well as globally asymptotically stable for *R*_0_ > 1. Numerical results are presented for the justifications of theoratical results.

## 1. Introduction

Mathematical modeling for disease transmission in host population is of great practical value in predicting and controlling disease spread (West Nile virus in North America in the 1990s, Avian influenza worldwide in the 2000s, SARS in Asia in 2003, etc.). The battle between infectious diseases and humans was heavily lopsided for much of the history. Since the pioneering work of Edward Jenner (a doctor, who worked in Gloucestershire, UK, noticed that individuals who had contracted cowpox rarely caught smallpox) on smallpox [1], the process of protecting individuals from infection by vaccination has become a routine, with substantial historical success in reducing both morbidity and mortality (see [2, 3] and references cited therein). Typically, after the initial infection, the host remains in a latent stage for a period of time before becoming infectious. For some diseases, the latent period is neither short nor negligible compared with the infectious period (scarlet fever: 1-2 days versus 14–21 days [4]; measles: 4–12 days versus 17–31 days [5]), leptospirosis, 2–12 days.

In this paper, we consider an epidemic model of vector-host population. The disease spread due to vector, for example, leptospirosis, dengue, malaria, west Nile virus, and so forth, is considered. We assume that the individuals after some time become susceptible again. Therefore, the term *λ*_*h*_ is added in the model. The model consists of the interaction of human and vector. The human population is divided in three subclasses, that is, susceptible human *S*_*h*_(*t*), infected human *I*_*h*_(*t*), and recovered human *R*_*h*_(*t*). The total population size of human is shown by *N*_1_ and *N*_1_ = *S*_*h*_(*t*) + *I*_*h*_(*t*) + *R*_*h*_(*t*). The vector population is divided in two subclasses, susceptible vector *S*_*v*_(*t*) and *I*_*v*_(*t*). The total size of the vector population is denoted by *N*_2_, with *N*_2_ = *S*_*v*_(*t*) + *I*_*v*_(*t*). The disease spread from vector, like leptospirosis, effects humans as well as cattle [9]. The human are infected by means of drinking water contaminated by dead rats or by infectious cattle while drinking water. This infection can also spread through the urine of infected human. Those who work in the fields, like marshy places, rice planters, going in dirty water, those who swimming in water are mostly infected. Weil's first time describes leptospirosis as a unique disease process in 1886, while 30 years before Inada and his colleagues identified the causal organism. The symptoms of leptospirosis are high fever, headache, chills, muscle aches, conjunctivitis (red eyes), diarrhea, vomiting, and kidney or liver problems (which may also include jaundice), anemia, and, sometimes, rash. Symptoms may last from a few days and up to several weeks. Deaths from this disease may occur but they are rare. For some cases, the infections can be mild and without obvious symptoms [10–14].

Many works have been done on vector-host models, as in [15–17]. Reference [15] presented a mathematical model of vector host in which the population dynamics of an SIR vector transmitted disease with two pathogen strains. They discussed the stability of the vector-host model and also presented the numerical simulation of their model. Reference [16], they presented the vector-host model of dengue disease; they analyzed the dengue model and presented their stability and numerical results for vector host dengue model. Reference [17], they presented a mathematical model in the form of demographic stochasticity and heterogeneity in transmission of infection dynamics of host-vector disease systems. Mathematical and theoretical discussion is presented in the paper. For more discussion, we refer the readers to the previously mentioned articles. In our models, we have presented the vector-host model with their stability analysis. We obtain, if *R*_0_ ≤ 1, it recover the community. However, for *R*_0_ > 1 the disease remains in the community. We present the global stability of the model and also we present in a good way the numerical simulation of the proposed model, choosing the different values for the parameters.

Many models have been proposed to represent the dynamics of both human and vector population [18–20]. Pongsuumpun et al. [21] developed mathematical models to study the behavior of leptospirosis disease. They represent the rate of change for both rats and human population. The human population are further divided into two main groups juveniles and adults. Triampo et al. [7] considered a deterministic model for the transmission of leptospirosis disease [7]. In their work, they considered a number of leptospirosis infections in Thailand and shown the numerical simulations. Zaman [6] considered the real data presented in [7] to study the dynamical behavior and role of optimal control theory. The dynamical interaction including local and global stability of leptospirosis infected vector and human population which can be found in Zaman et al. [22]. In their work they also presented the bifurcation analysis and presented the numerical simulations for different values of infection rate.

The structure of the paper is organized as follows.  Section 2 is devoted to the formulation of the mathematic model and reducing it to the normalized model. In Section 3, we present the infection-free equilibrium, the basic reproduction and the local and global stability of infection equilibrium. In Section 4, we present the disease persistence and existence of the endemic equilibrium. In Section 5, we show the local as well as the global stability of the endemic equilibrium for the reproduction number *R*_0_ > 1. The numerical results, conclusion and references are presented in Section 6. 

## 2. Mathematical Model

In this section, a vector-host epidemic model with direct transmission is presented. The host population at time *t* is divided into susceptible *S*_*h*_(*t*), *I*_*h*_(*t*) infected, and recovered *R*_*h*_(*t*) individuals. The vector population at time *t* is divided into susceptible *S*_*v*_(*t*) and infected vector population *I*_*v*_(*t*). The total population of humans is denoted by *N*_1_, and the total population of the vector is denoted by *N*_2_. Thus, *N*_1_(*t*) = *S*_*h*_(*t*) + *I*_*h*_(*t*) + *R*_*h*_(*t*) and *N*_2_(*t*) = *S*_*v*_(*t*) + *I*_*v*_(*t*). The mathematical representation of the model which consists of the system of nonlinear differential equations with five state variables is given by

(1) dShdt=ΛhN1−μhSh−β2ShIvN2−β1ShIhN1+λhRh,dIhdt=β2ShIvN2+β1ShIhN1−μhIh−δhIh−γhIh,dRhdt=γhIh−μhRh−λhRh,dSvdt=ΛvN2−γvSv−β3SvIhN1,dIvdt=β3SvIhN1−γvIv.

Here, Λ_*h*_ is the recruitment rate of human population; susceptible human can be infected by two ways of transmission, that is, directly, or through infected individuals; *β*_1_, *β*_2_ are the mediate transmission coefficients. *μ*_*h*_ is the natural mortality rate for humans; *γ*_*h*_ is the recovery rate for humans from the infections. We assumed that the disease may be fatal to some infectious hosts, so disease-related death rate from infected class occurs at human populations at *δ*_*h*_. The immune human once again susceptible at constant rate *λ*_*h*_, for some disease like dengue, the chances for susceptibility are less compared to dengue, West Nile virus, malaria, and so forth. Λ_*v*_ is the recruitment rate for vector population. The death rate of vector *γ*_*v*_, *β*_3_ is the disease carrying the vector to the host per unit time:

(2) $$\begin{array}{c} \frac{dN_{1}}{dt}=\Lambda_{h}N_{1}-\mu_{h}N_{1}-\delta_{h}I_{h}. \end{array}$$

### 2.1. Normalized Model

For the normalization of the model, we let ${\hat{S}}_{h}={\hat{S}}_{h}/N_{1}$, ${\hat{I}}_{h}=I_{h}/N_{1}$, ${\hat{R}}_{h}=R_{h}/N_{1}$, ${\hat{S}}_{v}=S_{v}/N_{2}$, and ${\hat{I}}_{v}={\hat{I}}_{v}/N_{2}$. It is easy to verify that ${\hat{S}}_{h}$, ${\hat{I}}_{h}$, ${\hat{R}}_{h}$, ${\hat{S}}_{v}$, and ${\hat{I}}_{v}$ satisfy the following system of differential equations:

(3) dS^hdt=Λh(1−S^h)+δhS^hI^h−β2S^hI^v−β1S^hI^h+λh(1−S^h−I^h),dI^hdt=β2S^hI^v+β1S^hI^h−(Λh+δh+γh)I^h+δhI^h2,dR^hdt=γhI^h−(Λh+λh)R^h+δhR^hI^h,dS^vdt=Λv(1−S^v)−β3S^vI^h,dIvdt=β3S^vI^h−γvI^v.

With restriction, ${\hat{S}}_{h}+{\hat{I}}_{h}+{\hat{R}}_{h}=N_{1}=1$, ${\hat{S}}_{v}+{\hat{I}}_{v}=N_{2}=1$ and ${\hat{I}}_{v}=1-{\hat{S}}_{v}$. In the first equation of the normalized model, we substituted ${\hat{R}}_{h}=1-{\hat{S}}_{h}-{\hat{I}}_{h}$. So in the normalized system the ${\hat{R}}_{h}$ does not appear. We reduced to the normalized model (3), and we will study the reduced model:

(4) dS^hdt=Λh(1−S^h)−β2S^hI^v+δhS^hI^h−β1S^hI^h+λh(1−S^h−I^h),dI^hdt=β2S^hI^v+β1S^hI^h−(Λh+δh+γh)I^h+δhI^h2dIvdt=β3(1−I^v)I^h−γvI^v.

We determine ${\hat{S}}_{v}$ and ${\hat{R}}_{h}$ from ${\hat{S}}_{v}=1-{\hat{I}}_{v}$ and ${\hat{R}}_{h}=1-{\hat{S}}_{h}-{\hat{I}}_{h}$, respectively. For reduced system (4), the feasible region is

(5) $$\begin{array}{c} \Omega :=\left\{\left({\hat{S}}_{h},{\hat{I}}_{h},{\hat{I}}_{v}\right)\in R_{+}^{3}\;|\;0\leq {\hat{S}}_{h}+{\hat{I}}_{h}+{\hat{I}}_{v}\leq 1\right\}. \end{array}$$

With the nonnegative initial conditions values of *Ω*, the system is positively invariant, and the proof is easy. 

## 3. Infection-Free Equilibrium and Basic Reproduction Number

 The basic reproduction for the reduced system (4) is given by

(6) $$\begin{array}{c} R_{0}=\frac{\beta_{1}}{\left(\Lambda_{h}+\delta_{h}+\gamma_{h}\right)}+\frac{\beta_{2}\beta_{3}}{\gamma_{v}\left(\Lambda_{h}+\delta_{h}+\gamma_{h}\right)}. \end{array}$$

The disease eradicated from the population by two ways, first with the varying size in population and ${\hat{I}}_{h}\to 0$, and the second one is *I*_*h*_ → 0, for detail see [23, 24]. We are thus inspired to seek the conditions for infection-free and endemic equilibrium. The infection-free equilibrium point for model (4) is $E_{o}=({\hat{S}}_{h}={\hat{S}}_{h}^{0},0,0)$ and for endemic equilibrium $E^{*}=({\hat{S}}_{h}^{*},{\hat{I}}_{h}^{*},{\hat{S}}_{v}^{*})$. The infection-free equilibrium is obtained by setting the left side of the reduced model (4), we obtain ${\hat{S}}_{h}^{0}=1$. Obviously the infection-free equilibrium $E_{o}=({\hat{S}}_{h}={\hat{S}}_{h}^{0}=1,0,0)$ belongs to *Ω* of reduced model (4), which exists for all positive parameters. Next, we prove the infection-free local asymptotical stability of model (4) at the arbitrary point $E_{1}=({\hat{S}}_{h},{\hat{I}}_{h},{\hat{I}}_{v})$.

**Theorem 1**

The infection-free equilibrium of reduced model (4) is stable locally asymptotically stable for *R*_0_ < 1 when *γ*_*v*_ + *C*_1_ > *β*_1_ and unstable for *R*_0_ ≥ 1.

**Proof**

The Jacobean matrix of the reduced model about the equilibrium point *E*_0_ is given by

(7) J(E0)=(−β2I^v−β1I^h−Λh+δI^h(δh−β1)S^h−λh−β2S^hβ2I^v+β1I^hβ1S^h−C1+2δhI^hβ2S^h0β3(1−I^v)−β3I^h−γv),

where *C*_1_ = Λ_*h*_ + *γ*_*h*_ + *δ*_*h*_.

The characteristics equation of the Jacobian matrix *J*(*E* ^0^) is obtained by

(8) $$\begin{array}{c} \left(-\Lambda_{h}-\lambda \right)\left(\left(\left(\beta_{1}-C_{1}\right)-\lambda \right)\left(\gamma_{v}-\lambda \right)-\beta_{2}\beta_{3}\right)=0. \end{array}$$

The eigenvalue −*λ*_*h*_ has a negative real part, and the rest of the two eigenvalues is calculated by Routh-Harwitz-Criteria. We write

(9) $$\begin{array}{c} \lambda^{2}+\lambda \left(\gamma_{v}+C_{1}-\beta_{1}\right)+\gamma_{v}C_{1}\left(1-R_{0}\right)=0, \end{array}$$

when *R*_0_ < 1, then Routh-Hurtwiz Criteria are satisfied if *γ*_*v*_ + *C*_1_ > *β*_1_. The infection-free equilibrium is locally asymptotically stable.

Next, we show the global asymptotical stability of infection-free equilibrium, by defining the Lyapunove function.

**Theorem 2**

If the threshold quantity *R*_0_ ≤ 1, the infection-free equilibrium of the reduced model (4) is globally asymptotically stable and is an unstable infection-free equilibrium for system (4), when *R*_0_ > 1.

**Proof**

To show the global stability of infection-free equilibrium of reduced model (4), we define the Lyapunove function in the following:

(10) $$\begin{array}{c} P\left(t\right)=\gamma_{v}{\hat{I}}_{h}+\beta_{2}{\hat{I}}_{v}. \end{array}$$

Taking the time derivative of (10), along the solution of system (4), we obtain

(11) P′(t)=γv[β2S^hI^v+β1S^hI^h−(Λh+δh+γh)I^h+δhI^h2]+β2[β3(1−I^v)I^h−γvI^v].

Using ${\hat{S}}_{h}=1-{\hat{I}}_{h}$ and simplifying, we get

(12) P′(t)=−γvβ2I^vI^h−γv(β1−δh)I^h2−β2β3I^hI^v−γv(Λh+γh+δh)(1−R0)I^h.

When *R*_0_ ≤ 1, the infection free-equilibrium is globally asymptotically stable, and *P*′(*t*) is negative. *P*′(*t*) becomes zero when ${\hat{I}}_{h}$ is zero and vice versa. By the Lasalle invariant principle [25], which implies that the infection-free equilibrium at the point *E*_0_ is globally asymptotically stable in *Ω*.

## 4. Disease Persistence

In this section, we study the uniform persistence of the reduced system (4). The disease persistence occurs for the case when the threshold parameter *R*_0_ > 1, by applying the acyclicity Theorem [26].

**Definition 3**

The reduced model (4) is called uniformly persistence if there exists a constant *c* ∈ (0,1) such that any solution $\left({\hat{S}}_{h},{\hat{I}}_{h},{\hat{I}}_{v}\right)$ with $\left({\hat{S}}_{h}(0),{\hat{I}}_{h}(0),{\hat{I}}_{v}(0)\right)$∈*Ω* satisfies

(13) $$\begin{array}{c} \min\left\{\underset{t\to \infty }{\mathrm{liminf}}{\hat{S}}_{h}\left(t\right),\underset{t\to \infty }{\mathrm{liminf}}{\hat{I}}_{h}\left(t\right),\underset{t\to \infty }{\mathrm{liminf}}{\hat{I}}_{v}\left(t\right)\right\}\geq c. \end{array}$$

Let *X* be a locally compact metric space with metric *d*, and let *C* be a closed nonempty subset of *X* with boundary ∂*Ω* and interior of *Ω* ^*o*^. Obviously, ∂*Ω* is the closed subset of *Ω*. Suppose that *ϕ*_*t*_(*x*) be a dynamical system defined on *Ω*. A subset *B* in *X* is said to be invariant if *ϕ*(*B*, *t*) = *B*. Define *T*_∂_ : = {*x* ∈ ∂*Ω* : *ϕ*_*t*_(*x*)∈∂*Ω*, for  all  *t* ≥ 0}.

**Lemma 4**

Assume that

(*H*_1_)   
*ϕ*(*t*) has a global attractor;
(*H*_2_) there exists an *N* = *N*_1_,…, *N*_*k*_ of pair-wise disjoint, compact, and isolated invariant set on ∂*Ω* such that;
(*a*_1_)   
*V*_*x*∈∂*Ω*_ ⊂ *V*_*j*=1_^*k*^ *N*_*j*_;
(*a*_2_)   no subset of *N* forming a cycle on ∂*Ω*;
(*a*_3_) each of *N*_*j*_ is also isolated in ∂*Ω*;
(*a*_4_)   
$W^{s}(N_{j})\cap \Omega^{o}=\hat{\phi }$ for every 1 ≤ *j* ≤ *k*, where *W* ^*s*^(*N*_*j*_) is the stable manifold of *N*_*j*_. Then *ϕ*(*t*) is uniformly persistent with respect to *Ω* ^*o*^ [26].

By the application of Lemma 4 to our model, suppose that

(14) $$\begin{array}{c} \Omega :=\left\{\left({\hat{S}}_{h},{\hat{I}}_{h},{\hat{I}}_{v}\right)\in R_{+}^{3}\;|\;0\leq {\hat{S}}_{h}+{\hat{I}}_{h}+{\hat{I}}_{v}\leq 1\right\}, \end{array}$$

from (5),

(15) $$\begin{array}{c} \Omega^{o}:=\left\{{\hat{S}}_{h},{\hat{I}}_{h},{\hat{I}}_{v}\in E,{\hat{I}}_{h},{\hat{I}}_{v}>0\right\},\;\;\partial \Omega =\frac{\Omega }{\Omega^{o}}. \end{array}$$

Clearly, *N*_∂_ = ∂*Ω*.

Hypotheisis (*a*_1_) and (*a*_2_) hold, for (4), reducing to ${\hat{S}}_{h}^{\prime }=(\Lambda_{h}+\lambda_{h})-(\Lambda_{h}+\lambda_{h}){\hat{S}}_{h}$, when *t*_*∞*_, then ${\hat{S}}_{h}^{\prime }(t)=1$. When *R*_*o*_ > 1, the infection-free equilibrium is unstable. Also, *W* ^*s*^(*N*) = ∂*Ω*. (*a*_3_) and (*a*_4_) are satisfied. Due to the boundedness the reduced system (4) always admits a global attractor, so *H*_1_ is satisfied. We now state the above discussion in the form of the following result.

**Theorem 5**

For *R*_0_ > 1, the reduced system (4), is uniformly persistent.

### 4.1. Existence of the Endemic Equilibrium

We have proved in Section 4 the local asymptotical stability of infection-free equilibrium when *R*_0_ < 1. In such case, when the infection-free equilibrium is locally asymptotically stable for is *R*_0_ < 1, the disease dies out and no endemic equilibrium exists. From epidemiological point of view, it is important to show the existence of endemic equilibrium when *R*_0_ > 1.

Let $E^{*}=({\hat{S}}_{h}^{*},{\hat{I}}_{h}^{*},{\hat{I}}_{v}^{*})$ belong to *Ω* which is an endemic equilibrium. From reduced system (4), its coordinates should satisfy

(16) Λh(1−S^h∗)−β2S^h∗I^v∗+δhS^hI^h∗−β1S^h∗I^h∗ +λh(1−S^h∗−I^h∗)=0,β2S^h∗I^v∗+β1S^h∗I^h∗−(Λh+δh+γh)I^h∗+δhI^h∗2=0,β3(1−I^v∗)I^h∗−γvI^v∗=0,

with ${\hat{S}}_{h}^{*}>0$, ${\hat{I}}_{h}^{*}>0$, and ${\hat{I}}_{v}^{*}>0$. By adding the system (16), and solve for ${\hat{I}}_{h}^{*}$, we obtain

(17) ((Λh+λh)−δhI^h∗)(1−S^h∗−I^h∗)  =−(γv+γh)+(β3I^h∗−γv)(1−I^v∗).

This gives the range for ${\hat{I}}_{h}^{*}$ in the following:

(18) $$\begin{array}{c} 0<{\hat{I}}_{h}^{*}<\left(\left\{1,\min\frac{\left(\Lambda_{h}+\lambda_{h}\right)}{\delta_{h}}\right\},\left\{1,\min\left\{\frac{\beta_{3}}{\gamma_{v}}\right\}\right\}\right). \end{array}$$

From (18), note that the disease-related death *δ*, less than the (Λ_*h*_ + *λ*_*h*_), the birth rate Λ, the rate at which the human become susceptible *λ*_*h*_, the sum of (Λ_*h*_ + *λ*_*h*_), and the contact rate coefficient *β*_3_, and the less value of *γ*_*v*_ (natural death rate of vector) will lie in the interval (0,1). Now, further eliminate ${\hat{S}}_{h}^{*}$ and ${\hat{I}}_{v}^{*}$ from (10), then ${\hat{I}}_{h}^{*}$ satisfies

(19) ((Λh+λh)−γhI^h∗)−[(λh+δh+γh)−δhI^h∗]I^h∗=(Λh+λh−δhI^h∗){(Λh+δh+γh)−δhI^h∗β2β3+β1(γv+β3I^h∗)(γv+β3I^h∗)}.

Further simplification gives

(20) $$\begin{array}{c} f\left({\hat{I}}_{h}^{*}\right)={\hat{I}}_{h}^{*3}+B_{1}{\hat{I}}_{h}^{*2}+B_{2}{\hat{I}}_{h}^{*}+B_{3}, \end{array}$$

where

(21) B1=β1γvδh+β3δh(β2+Λh+γh)+β3[β1(2γh+λh+δh)−(Λh+λh)(Λh+γh)]β1β3δh,B2=β1γv(2γh+λ−h+δh)+β1β3(Λh+λh)+(Λh+λh−δh)[δhγv+γv(Λh+δh+γh)]β1β3δh,B3=(Λh+γh+δh)(Λh+λh)γv(R0−1)β1β3δh,

and the equilibria of the reduced system (4) is given by

(22) $$\begin{array}{c} S_{h}^{*}=\frac{\left(\left(\Lambda_{h}+\lambda_{h}\right)-\lambda_{h}{\hat{I}}_{h}^{*}\right)\left(\beta_{3}{\hat{I}}_{h}^{*}+\gamma_{v}\right)}{\left(\beta_{2}\beta_{3}{\hat{I}}_{h}^{*}+\left[\left(\Lambda_{h}+\lambda_{h}\right)+{\hat{I}}_{h}^{*}\left(\beta_{1}-\delta_{h}\right)\right]\left(\beta_{3}I_{h}+\gamma_{v}\right)\right)},\\ I_{v}^{*}=\frac{\beta_{3}I_{h}}{\beta_{3}I_{h}+\gamma_{v}}. \end{array}$$

The positive endemic equilibrium depends upon $\left(\Lambda_{h}+\lambda_{h}\right)>\lambda_{h}{\hat{I}}_{h}^{*}$ and *β*_1_ ≥ *δ*_*h*_ when *R*_0_ > 1 we get a positive endemic equilibrium point. We now state the above in the following result.

**Theorem 6**

When *R*_0_ > 1, a unique positive endemic equilibrium exists for reduced system (4), In other case the existence of disease-free equilibrium.

## 5. Global Stability of Endemic Equilibrium

**Theorem**

For *R*_0_ > 1, the reduced model (4), about the endemic equilibrium point E*, is globally asymptotically stable, and unstable for *R*_0_ > 1.

**Proof**

To prove that the reduced model (4) is globally asymptotically stable, we obtain the Jacobian matrix J* about E* which is given by

(23) $$\begin{array}{c} J^{*}\left(E^{*}\right)=\left(\begin{array}{ccc} -\Lambda_{h}-\beta_{2}{\hat{I}}_{v}+\delta_{h}{\hat{I}}_{h}-\beta_{1}{\hat{I}}_{h}-\lambda_{h} & \delta_{h}{\hat{S}}_{h}-\beta_{1}{\hat{S}}_{h}-\lambda_{h} & -\beta_{2}{\hat{S}}_{h}\\ \beta_{2}{\hat{I}}_{v}+\beta_{1}{\hat{I}}_{h} & \beta_{1}{\hat{S}}_{h}-\left(\Lambda_{h}+\delta_{h}+\gamma_{h}\right)+2\delta_{h}{\hat{I}}_{h} & \beta_{2}{\hat{S}}_{h}\\ 0 & \beta_{3}\left(1-{\hat{I}}_{v}\right) & -\beta_{3}{\hat{I}}_{h}-\gamma_{v} \end{array}\right). \end{array}$$

The second additive compound matrix for J*(E*) is given in the following. Also see, the Appendix for the second additive compound matrix.

(24) J[2](E∗)=(A11β2S^hβ2S^hβ3(1−I^v)A22−β1S^h+δhS^h−λh0β2I^v+β1I^hA33),

where

(25) A11=−Λh−β2I^v+δhI^h−β1I^h−λh+β1S^h−(Λh+δh+γh)+2δhI^h,A22=−Λh−β2I^v+δhI^h−β1I^h−λh−β3I^h−γv,A33=β1S^h−(Λh+δh+γh)+2δhI^h−β3I^h−γv,P=P(S^h,I^h,I^v)=diag⁡(1,I^vI^h,I^vI^h),

where

(26) $$\begin{array}{c} P^{-1}=\mathrm{diag}\left(1,\frac{{\hat{I}}_{h}}{{\hat{I}}_{v}},\frac{{\hat{I}}_{h}}{{\hat{I}}_{v}}\right),\\ P_{f}=\mathrm{diag}\left(0,\frac{{\hat{I}}_{v}{\hat{I}}_{h}^{\prime }-{\hat{I}}_{v}^{\prime }{\hat{I}}_{h}}{{{\hat{I}}_{h}}^{2}},\frac{{\hat{I}}_{v}{\hat{I}}_{h}^{\prime }-{\hat{I}}_{v}^{\prime }{\hat{I}}_{h}}{{{\hat{I}}_{h}}^{2}}\right). \end{array}$$

And *P*_*f*_ *P* ^−1^ is

(27) $$\begin{array}{c} P_{f}P^{-1}=\mathrm{diag}\left(0,\frac{{\hat{I}}_{h}^{\prime }}{{\hat{I}}_{h}}-\frac{{\hat{I}}_{v}^{\prime }}{{\hat{I}}_{v}},\frac{{\hat{I}}_{h}^{\prime }}{{\hat{I}}_{h}}-\frac{{\hat{I}}_{v}^{\prime }}{{\hat{I}}_{v}}\right). \end{array}$$

And *P*_*f*_ *J* ^[2]^ *P* ^−1^ is

(28) PfJ[2]P−1 =J[2] =(A11β2S^hβ2S^hβ3(1−I^v)A22−β1S^h+δhS^h−λh0β2I^v+β1I^hA33).

So we write

(29) $$\begin{array}{c} \hat{B}=P_{f}P^{-1}+P_{f}J^{\left[2\right]}P^{-1}=\left(\begin{array}{cc} {\hat{B}}_{11} & {\hat{B}}_{12}\\ {\hat{B}}_{21} & {\hat{B}}_{22} \end{array}\right), \end{array}$$

where

(30) B^11=−Λh−β2I^v+δhI^h−β1I^h−λh   +β1S^h−(Λh+δh+γh)+2δhI^h,B^12=(β2S^h,β2S^h),  B^21=(β3(1−I^v),0)T,B^22=(A22+I^h′I^h−I^v′I^v−β1S^h+δhS^h−λh0I^h′I^h−I^v′I^v+A33).

Suppose that the vector $\left(\hat{u},\hat{v},\hat{w}\right)$ in *R* ^3^ and its norm ||·|| will be defined as

(31) $$\begin{array}{c} ||\left(\hat{u},\hat{v},\hat{w}\right)||=\max\left\{\left|\hat{u}\right|,\left|\hat{v}\right|+\left|\hat{w}\right|\right\}. \end{array}$$

Suppose that $\mu \hat{B}$ represents Lozinski measure with the previously defined norm. So, as described in [27], we choose

(32) $$\begin{array}{c} \mu \left(\hat{B}\right)\leq \sup\left(g_{1},g_{2}\right), \end{array}$$

where

(33) $$\begin{array}{c} g_{1}=\mu_{1}\left({\hat{B}}_{11}\right)+\left|{\hat{B}}_{12}\right|,\;\;g_{2}=\left|{\hat{B}}_{21}\right|+\mu_{2}\left({\hat{B}}_{22}\right). \end{array}$$

$\left|{\hat{B}}_{21}\right|$ and $\left|{\hat{B}}_{12}\right|$ are the matrix norm with respect to vector *ℓ*, and *μ*_1_ represents the Lozinski measure with respect to this *ℓ* norm, then

(34) μ1(B^11)=−Λh−β2I^v+δhI^h−β1I^h−λh+β1S^h−(Λh+δh+γh)+2δhI^h,|B^21|=|β3(1−I^v)|,|B^12|=max⁡⁡{β2S^h,β2S^h}=β2S^h,μ1(B^22)=max⁡⁡{I^h′I^h−I^v′I^v+A22,I^h′I^h−I^v′I^v+A33}∴g1=μ1(B^11)+|B^12|=−(Λh+λh)−β2I^v−β1I^h+δhI^h+δhI^h+β2S^h+2δhI^h−(Λh+γh+γh)=−(Λh+λh)−β2I^v−β1I^h+β2S^h+2δhI^h+I^h′I^h−β2S^hI^vI^h−β1S^h.

Use

(35) $$\begin{array}{c} \frac{{\hat{I}}_{h}^{\prime }}{{\hat{I}}_{h}}-\beta_{2}{\hat{S}}_{h}\frac{{\hat{I}}_{v}}{{\hat{I}}_{h}}-\beta_{1}{\hat{S}}_{h}=-\left(\Lambda_{h}+\gamma_{h}+\gamma_{h}\right)+\delta_{h}{\hat{I}}_{h}. \end{array}$$

From system (4) and equation (2),

(36) g1≤I^h′I^h−(Λh+λh)−I^h(β1−2δh),g2=|B^21|+μ1(B^22),=β3(1−I^v)+I^h′I^h−I^v′I^v+A22+A33=β3(1−I^v)+I^h′I^h−I^v′I^v−Λh−β2I^v+δhI^h−β1I^h−λh−β3I^h+β1S^h−(Λh+δh+γh)+2δhI^h−β3I^h−γv.

Using

(37) $$\begin{array}{c} \frac{{\hat{I}}_{v}^{\prime }}{I_{v}}=\beta_{3}{\hat{S}}_{h}\frac{{\hat{I}}_{h}}{I_{v}}-\gamma_{v}. \end{array}$$

From the third equation of system (4),

(38) $$\begin{array}{c} g_{2}\leq \frac{{\hat{I}}_{h}^{\prime }}{{\hat{I}}_{h}}-\left(\Lambda_{h}+\lambda_{h}\right)-\left(\beta_{1}-2\delta_{h}\right){\hat{I}}_{h}. \end{array}$$

So,

(39) $$\begin{array}{c} \mu_{1}\hat{B}\leq \sup\left(g_{1},g_{2}\right)\leq \frac{{\hat{I}}_{h}^{\prime }}{{\hat{I}}_{h}}-\beta ,\;\text{where}\;\;\beta =\left(\beta_{1}-2\delta_{h}\right), \end{array}$$

then

(40) $$\begin{array}{c} \frac{1}{t}\int_{0}^{t}\mu_{1}\hat{B}ds\leq \frac{1}{t}\int_{0}^{t}\left(\frac{{\hat{I}}_{h}^{\prime }}{{\hat{I}}_{h}}-\beta \right)ds=\frac{1}{t}\ln\frac{{\hat{I}}_{h}^{\prime }\left(t\right)}{{\hat{I}}_{h}^{\prime }\left(0\right)}-\beta \end{array}$$

implies that *q* ≤ −*β*/2 < 0. Thus, the result [28] implies that the positive equilibrium point of E* is globally asymptotically stable.

## 6. Numerical Simulations and Conclusion

**Figure 1:**
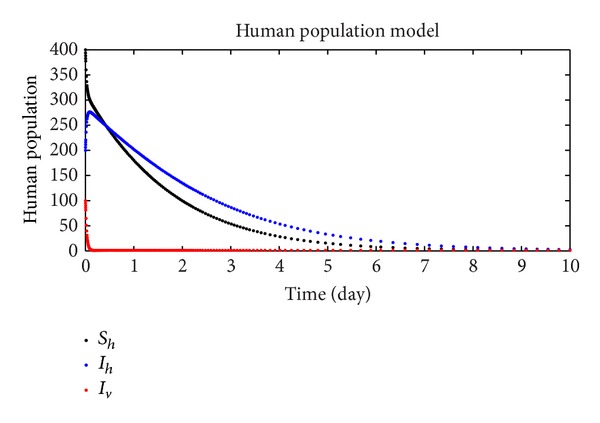
The plot shows the human population.

**Figure 2:**
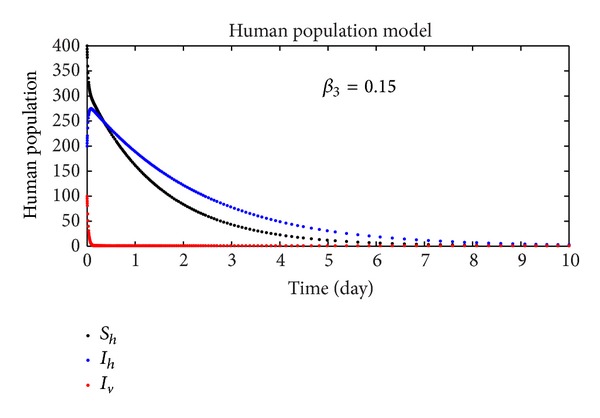
The plot shows the human population.

**Figure 3:**
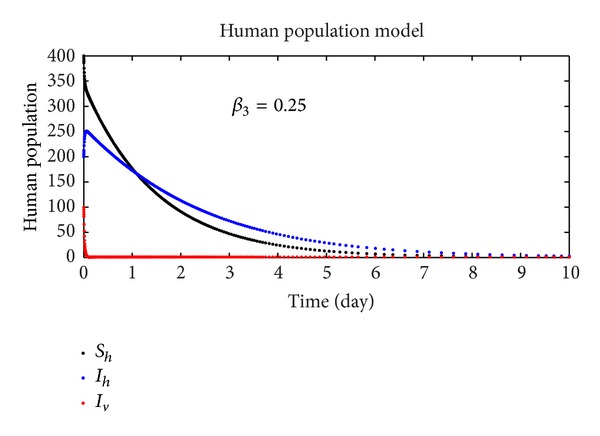
The plot shows the human population.

**Figure 4:**
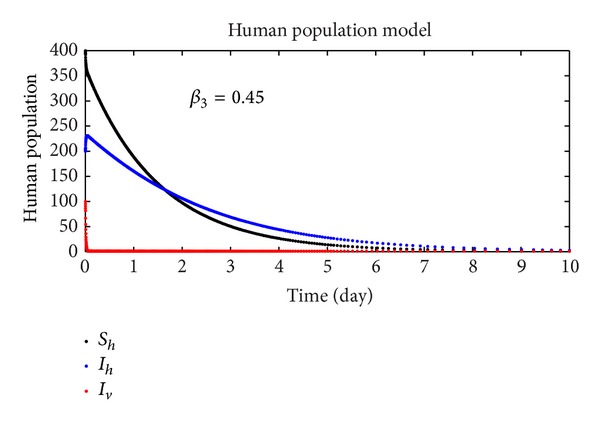
The plot shows the human population.

**Figure 5:**
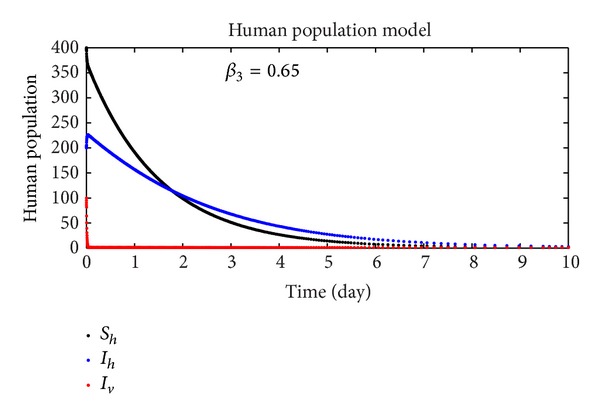
The plot shows the human population.

**Figure 6:**
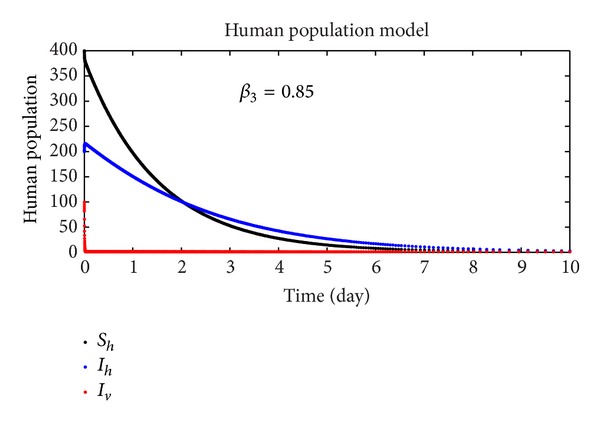
The plot shows the human population.

**Figure 7:**
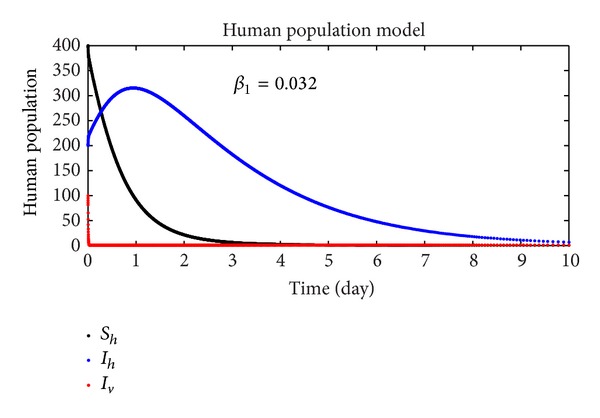
The plot shows the human population.

**Figure 8:**
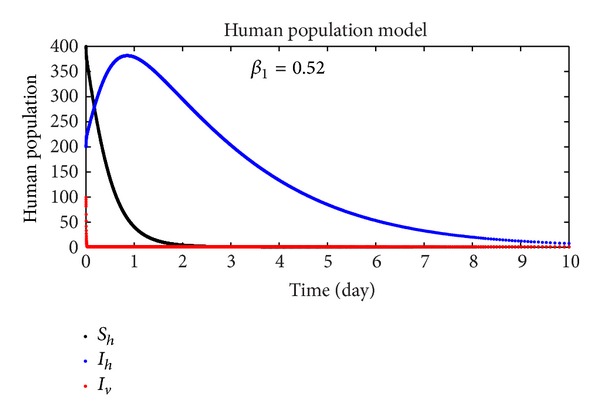
The plot shows the human population.

In this section, we discuss the numerical simulation of the reduced model (4), by using Runge-Kutta order four scheme. The model for different parameters and their numerical results are presented in Figures 1, 2, 3, 4, 5, 6, 7, and 8. The parameters and their values are presented in Table 1.  Figure 1 represents the population dynamics of model (4). Varying the parameters in Figures 2 to 6, we obtained different results. Increasing *β*_3_, the number of infected human increases. The variability of the population effects the individuals numbers (infected individuals). For changing the value of *β*_1_, we get different results in Figures 7 and 8. In this work, we have presented a mathematical model of vector-host disease like (leptospirosis, West Nile virus, dengue, etc.), which spreads through the vector, has been presented. The system is stable locally as well as globally about the disease-free equilibrium ${\hat{S}}_{h}^{o}=1,0,0$, when reproduction number *R*_0_ < 1, and the unstable equilibrium occurs for *R*_0_ ≥ 1. When the reproduction number *R*_0_ > 1, there exists persistence. The disease permanently exists in the community if *R*_0_ > 1. Then, we obtained the global stability of endemic equilibrium. The numerical simulations were presented for the illustration of theoretical results. 

## Figures and Tables

**Table 1:** Parameter values used in the numerical simulations of the model.

Notation	Parameter description	Value	Reference
Λ_*h*_	Recruitment rate for human	1.6	[6]
*λ*_*h*_	Proportionality constant	0.066	[7]
*μ*_*h*_	Natural death rate of human	4.6 × 10^−5^	[6]
*γ*_*v*_	Natural death rate of vector	1.8 × 10^−3^	[6]
*δ*_*h*_	Death rate due to disease at human class	1.0 × 10^−5^	[8]
*γ*_*h*_	Recovery rate of the infection	2.7 × 10^−3^	[8]
Λ_*v*_	Birth rate of vector	1.9 × 10^−3^	Assumed
*β*_2_	Transmission between *S*_*h*_ and *I*_*v*_	0.0089	Assumed
*β*_3_	Transmission between *S*_*v*_ and *I*_*h*_	0.0079	Assumed
*β*_1_	Transmission coefficient between *S*_*h*_ and *I*_*h*_	0.00013	Assumed
*γ*_*v*_	Natural death rate of vector	0.0027	[6]

## Appendix

Consider the following:

(A.1) $$\begin{array}{c} J^{\left[2\right]}=\left[\begin{array}{ccc} a_{11}+a_{22} & a_{23} & -a_{13}\\ a_{32} & a_{11}+a_{33} & a_{12}\\ -a_{31} & a_{21} & a_{22}+a_{33} \end{array}\right]. \end{array}$$
